# How does Tourette syndrome impact adolescents’ daily living? A text mining study

**DOI:** 10.1007/s00787-022-02116-1

**Published:** 2022-12-02

**Authors:** Cyril Atkinson-Clement, Marion Duflot, Eloise Lastennet, Leïla Patsalides, Emma Wasserman, Therese-Marie Sartoris, Clément Tarrano, Charlotte Rosso, Pierre Burbaud, Emmanuelle Deniau, Virginie Czernecki, Emmanuel Roze, Andreas Hartmann, Yulia Worbe

**Affiliations:** 1 Sorbonne Université, Paris Brain Institute Institut du Cerveau-ICM, CNRS, Hôpital de La Pitié Salpêtrière (DMU 6), InsermParis, AP-HP France; 2 Precision Imaging Beacon, School of Medicine, University of Nottingham, Nottingham, UK; 3 Urgences Cérébro-Vasculaires, Pitié-Salpétrière Hospital, Paris, France; 4 Centre Hospitalier Universitaire de Bordeaux, Institut des Maladies Neurodégénératives, CNRS, University of Bordeaux, Bordeaux, France; 5 National Reference Center for Tourette Syndrome, Assistance Publique des Hôpitaux de Paris, Groupe Hospitalier Pitié-Salpêtrière, 75013 Paris, France; 6 Department of Neurophysiology, Saint Antoine Hospital, Assistance Publique-Hôpitaux de Paris, Paris, France

**Keywords:** Daily living, School, Stigma, Text mining

## Abstract

**Abstract:**

Tourette syndrome is a neurodevelopmental disease in which clinical manifestations are essentially present during childhood and adolescence, corresponding to one of the critical development phases. However, its consequences on the daily lives of young patients have been insufficiently investigated. Here, we aimed to investigate this using a statistical text mining approach, allowing for the analysis of a large volume of free textual data. Sixty-two adolescents with Tourette syndrome participated in an interview in which they discussed their daily life (i) in school, (ii) at home, and (iii) with strangers, (iv) the aspect of Tourette syndrome which caused the most difficulty, and (v) their thoughts regarding their future as adults. Following data pre-processing, these corpora were analyzed separately using the IRAMUTEQ software through factorial correspondence analysis to identify the most commonly recurring topics of each corpus, and their relations with clinical features. The main difficulty corpus was directly related to comorbidities of Tourette syndrome. Daily life at home was correlated with executive functioning. Difficulties at school were related to a higher severity of tics. Thoughts regarding future daily life were worst for the youngest patients and were correlated with executive functioning and a higher depression score. Taken altogether, our results highlighted that social stigma was a pervasive topic among our corpora. From a clinical standpoint, tic severity was especially related to difficulties at school, while comorbidities had a high impact on social daily living and cost for managing both tics and symptoms of comorbidities.

**Trial registration:**

clinicaltrials.gov/ct2/show/NCT04179435.

**Supplementary Information:**

The online version contains supplementary material available at 10.1007/s00787-022-02116-1.

## Introduction

Tourette syndrome (TS) is a neurodevelopmental disease characterized by the presence of multiple chronic motor and vocal tics. In addition, TS is frequently associated with attention-deficit hyperactivity disorder (ADHD) and obsessive–compulsive disorder (OCD) as comorbid disorders. TS occurs during childhood and the peak of tic severity is generally reached around 10–12 years [1], which corresponds to the critical development phase of adolescence. While the clinical manifestations and pathophysiology of TS have been largely studied, the subjective experience of young individuals in general and during adolescence in particular has been poorly investigated.

In both children and adolescents with TS compared to healthy individuals, a decrease in quality of life in numerous domains of daily life was shown [2, 3]. This decreased quality of life could be related either to the severity of tics or to comorbidities [4–6]. For young individuals especially [3], this leads to alteration of social, school, and familial functioning [7, 8]. The social consequences of TS are also of major importance, since they lead to strong stigma and perceived stigma [9, 10]. In fact, approximately 41% of individuals with TS feel as though they are treated differently because of their tics [11] based on the attitudes adopted by those surrounding them (e.g., family, friends, and strangers). Altogether, most of the life domains seem to be affected, including the physical domain (due to the tics themselves), psycho-emotional functioning (e.g., stress due to tic expression), school/work functioning (e.g., stigmatization and social exclusion), and even familial functioning, since tics are mostly expressed at home [7, 12–14]. Therefore, TS may compromise children and adolescent patients’ schooling [15].

In this study, we aimed to give a voice to adolescents living with TS through an interview, and to report it using statistical text mining (i.e., convert unstructured natural language to structured database able to be statistically analyzed). This approach is promising, since, contrary to qualitative approaches, it allows for the analysis of a large volume of textual data. Indeed, for the overwhelming majority of research on psychiatric and neurological conditions, natural language data are not considered, leading to a lack of consideration of the patients’ lived and subjective experience. With the recent development of textual data mining, we can go beyond these limitations by examining a considerable volume of patients’ speech and correlating it with clinical outcomes using a rigorous and systematic approach.

We focused on three domains of our patients’ daily lives: school, family functioning, and interactions with strangers, as well as by considering the most crippling aspects of TS and the future expectations of our patients.

## Methods

### Subjects

Sixty-two adolescents with TS ranging from 13 to 18 years old were recruited through the Reference Center for Tourette Syndrome at the Pitié-Salpêtrière Hospital in Paris without any criteria of social or cultural origin. All patients at the age of majority (≥ 18 years) and all parents and patients under the age of majority (< 18 years) gave their written consent to participate in the study. The exclusion criteria were a lack of capacity or unwillingness to give consent for the study, evidence of either present or prior substance addiction, a past or present history of psychosis, and neurological disorder other than TS. This project was approved by the national ethics committee (2019.06.05 bis _19.04.26.51955).

All patients were assessed for tic severity using the Yale Global Tic Severity Scale (YGTSS [16]), OCD using the Yale-Brown Obsessive Compulsive Scale (Y-BOCS [17, 18]) and ADHD using the ADHD Self-Report Scale (ASRS; [19]). Additional self-reported assessments were performed by the patients for depression (Beck Depression Inventory [BDI] [20]), impulsivity (child version of the Urgency, Premeditation, Perseverance, Sensation Seeking, Impulsive Behavior Scale [UPPS] [21]), or their parents for executive functioning (Behavior Rating Inventory of Executive Function [BRIEF] [22]). All descriptive data and statistics are available in supplementary material.

### Data acquisition

The 62 individuals with TS participated in a short recorded interview (less than 15 min) which was performed in a quiet room with a minimum number of individuals (i.e., the patient, the interviewer, and eventually one additional person from the research team). Before the interview, patients were invited to develop their responses as much as possible and were encouraged to talk about different topics. The interviewer—a neuropsychologist (CAC)—remained the same for all patients. Particular attention was given to always formulate the question in the same manner and to take part as little as possible in the discussion to avoid interviewer bias.

The interview was built to discuss the following points: (i) the daily life of patients in school, at home, and with strangers; (ii) the aspect of TS which had the worst consequences on their daily functioning; (iii) their thoughts regarding their future daily life as adults. The questions were asked in a pseudo-randomized order. The patients were also invited to add any information they wanted to share.

### Data pre-processing

The interviews were first manually transcribed by four different members of the team (i.e., MD, EL, LP, and EW) and then divided by question, leading to the composition of five corpora. Before the corpora were statistically analyzed, a final reading was performed by CAC to homogenize several terms (to avoid the analyses considering two synonyms to be unrelated) and to add terms in the case of implicit reference.

Then, the corpora were loaded in the IRAMUTEQ software (R Interface for Multidimensional Analysis of Texts and Questionnaires, version 0.7–alpha 2), a text mining dedicated tool based on both R and Python languages [23]. IRAMUTEQ applies text pre-processing in three steps. First, it divides each text of the corpus into smaller units on the criteria of size and punctuation, called “*text segments*”. This segmentation has the advantage of decreasing the units’ granularity and increasing the precision of the analyses. Then, a lemmatization of the corpus was performed, grouping together the inflected forms of a word to their simplest form to analyze them as a single item (e.g., “*ate*” and “*eaten*” were changed for “*eat*”). Finally, words were categorized into two subcategories: “*full words*” (e.g., verbs, nouns, and adjectives) and “*tool words*” (e.g., pronouns and determents) to only consider full words in the analyses.

### Data analyses

We analyzed the five corpora separately. For each, we performed a factorial correspondence analysis (FCA) on each full word with a frequency ≥ 5, to measure the relationships between words of the corpus. This analysis was applied by building a binary table with all text segments as rows and all full words as columns, with the code “0” if the word is not present in the segment and “1” if the word is present. In other words, FCA is used to determine the proximity between words based on their frequencies. Furthermore, FCA identified a small number of independent dimensions (so-called “*factors*”) based on an analysis of inertia decomposition, which corresponds to the variance of our data (i.e., the full words). FCA is applied by first calculating the total variance of the data (called total inertia), then decomposing the whole data by finding the factor which explains the higher part of the variance (i.e., the factor (1), and then the second factor which explains another part of the variance (i.e., the factor (2) until the whole variance has been explained. This process is called inertia decomposition. The generated dimensions allow for a reduction of the variance of the raw data (i.e., the whole corpus) by simultaneously minimizing the distance between the profiles and axes and maximizing the amount of explained inertia. For example, the first factor of an FCA is the one which captures the largest part of variance of the corpus, followed by the second factor, etc. In other words, this analysis generates several factors, for which all full words will have a specific position, a specific correlation with the factor, and a specific contribution to the factor. As this approach allows for the reduction of a corpus’ complexity, we decided to consider only the two first factors (i.e., the most meaningful), and for these, we considered all words with a contribution higher than random to be significant (i.e., the contribution of specific words should be higher than the number of words implicated in the analysis divided by 100, e.g., if 100 words were involved in the analysis, the contribution should be higher than 1%). This selection allowed us, for each factor, to build a gradient from words significantly negatively correlated to the factor to words significantly positively correlated with the factor. To increase the readability of our results, we named each side of the two considered factors on the basis of the significant words as well as the most relevant text segments. Finally, for illustrative purpose, we reported, for each side of each factor, one phrase from the most typical individual with TS (i.e., from the patients with the lowest and the highest correlations with each factor). The supplementary Fig. 1 illustrates the FCA process.

Then, we extracted the correlation of each individual with TS with the two considered factors. These positions were used (1) to determine if most of the patients were more oriented toward the negative or the positive position of this factor using *χ*2 test. To do this, we built a contingency table with two columns to obtain the number of participants who had a negative or a positive score with regards to the factor. (2) To correlate them with demographic and clinical data. The variables considered were as follows: demographic data (i.e., gender and age), tic severity (i.e., YGTSS subscales), medication status, severity of comorbidities as assessed during the study (i.e., Y-BOCS and ASRS), impulsivity (i.e., UPPS), and depression (i.e., BDI) as perceived by the patients and executive functioning as perceived by the parents (i.e., BRIEF). For all analyses, the threshold for significance was set at *p* ≤ 0.05.

## Data sharing

The data that support the findings of this study are available from the corresponding author upon reasonable request.

## Results

### Corpus 1: main difficulty

**Fig. 1:**
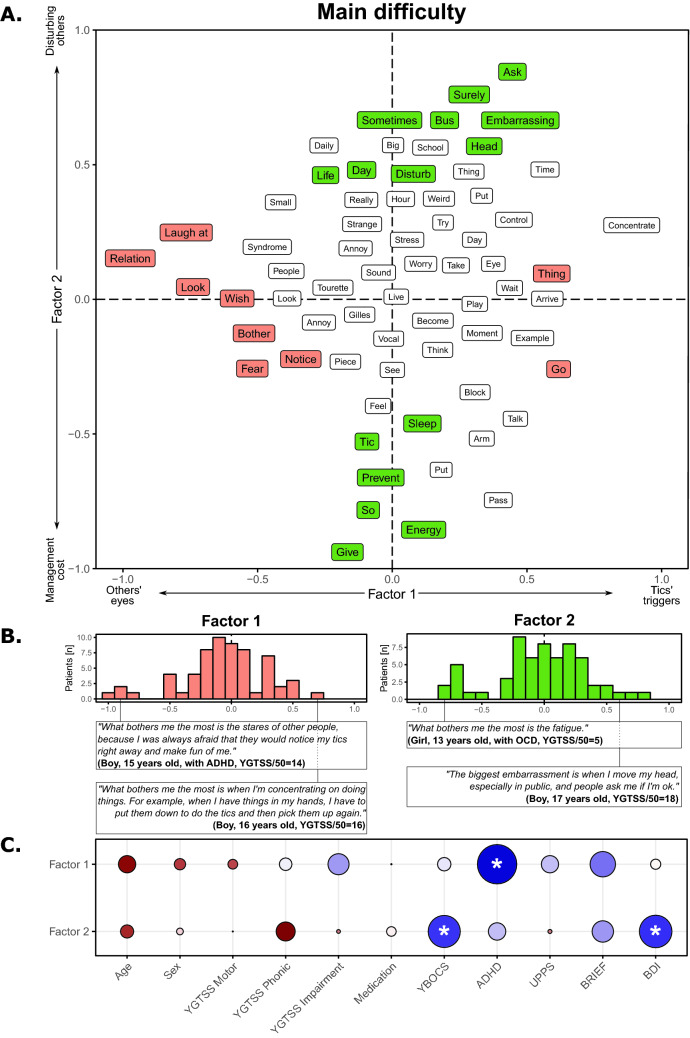
Results on the main difficulty corpus

The corpus on the main difficulty related to TS was composed of 6,498 words. The FCA’s first factor referred to difficulties ranging from external (i.e., others’ eyes; negative score) to internal issues (i.e., tics’ triggers; positive score; see Fig. 1A), with an equivalent TS distribution on this axis (i.e., equivalent number of patients with a positive and a negative score on this axis; *χ*_(1)_ = 1.03, *p* = 0.31; Fig. 1B). We found a significant negative correlation between this score and the degree of ADHD symptoms as measured with the ASRS (*t*_(60)_ = − 2.909, *p* = 0.005, *r* = − 0.351; Fig. 1C). The second factor also described, but differently, a range from internal (i.e., symptoms management’s cost; negative score) to external issues (i.e., the fear to disturb others; positive score; see Fig. 1A), with an equivalent TS distribution on the axis (*χ*_(1)_ = 0, *p* = 1; Fig. 1B). This factor was negatively correlated with the degree of OCD symptoms (*t*_(60)_ = − 2.36, *p* = 0.021, *r* = − 0.291) and with BDI score (*t*_(60)_ = − 2.395, *p* = 0.019, *r* = − 0.295; Fig. 1C).

Panel A shows the results of the factorial analysis for the first two factors. Words in orange correspond to words with a significant involvement in the first factor, while words in green correspond to words with a significant involvement in the second factor. Panel B highlights the position of the TS patients on the first factor (left) and on the second factor (right), and the sentences of the most typical individuals with TS. Panel C shows the correlations between the positions of factor 1 (top) and factor 2 (bottom) and the demographic and clinical variables. Red colors correspond to positive correlations, while blue colors correspond to negative correlations. The size of the point and the color intensity indicate the strength of the correlations. ‘*’ indicates a significant correlation. Note that a word could be present multiple times due to the translation from the original French results to English.

### Corpus 2: family life

**Fig. 2:**
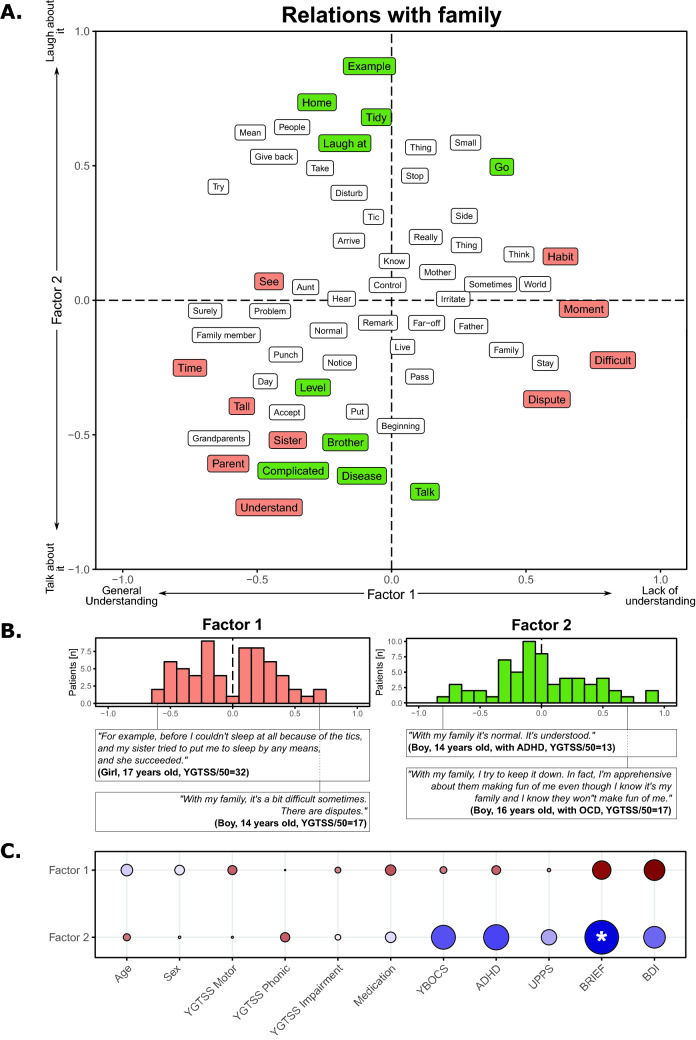
Results on the daily living at home corpus

The family life corpus was composed of 5872 words. The first FCA factor highlighted familial acceptance of the disease, ranging from a generally good understanding (negative score) to a lack of understanding (positive score; Fig. 2A) without asymmetrical distribution (*χ*_(1)_ = 0.06, *p* = 0.79; Fig. 2B) nor any significant demographic or clinical correlation (Fig. 2C). The second factor was focused on the integration of TS in daily life, ranging from an ability to discuss it with understanding (negative score) to having family members laugh about it (positive score; Fig. 2A), with an equivalent distribution between the two axes (*χ*_(1)_ = 2.332, *p* = 0.13; Fig. 2B). We found a negative correlation between the patients’ position on this axis and the executive functioning as assessed by the parents with the BRIEF score (*t*_(60)_ = − 2.488, *p* = 0.016, *r* = − 0.306; Fig. 2C).

Panel A shows the results of the factorial analysis for the first two factors. Words in orange correspond to words with a significant involvement on the first factor, while words in green correspond to words with a significant involvement on the second factor. Panel B highlights the position of the TS patients on the first factor (left) and on the second factor (right), and the sentences of the most typical individuals with TS. Panel C shows the correlations between the positions of factor 1 (top) and factor 2 (bottom) and the demographic and clinical variables. Red colors correspond to positive correlations, while blue colors correspond to negative correlations. The size of the point and the color intensity indicate the strength of the correlations. ‘*’ indicates a significant correlation. Note that a word could be present multiple times due to the translation from the original French results to English.

### Corpus 3: life at school

**Fig. 3:**
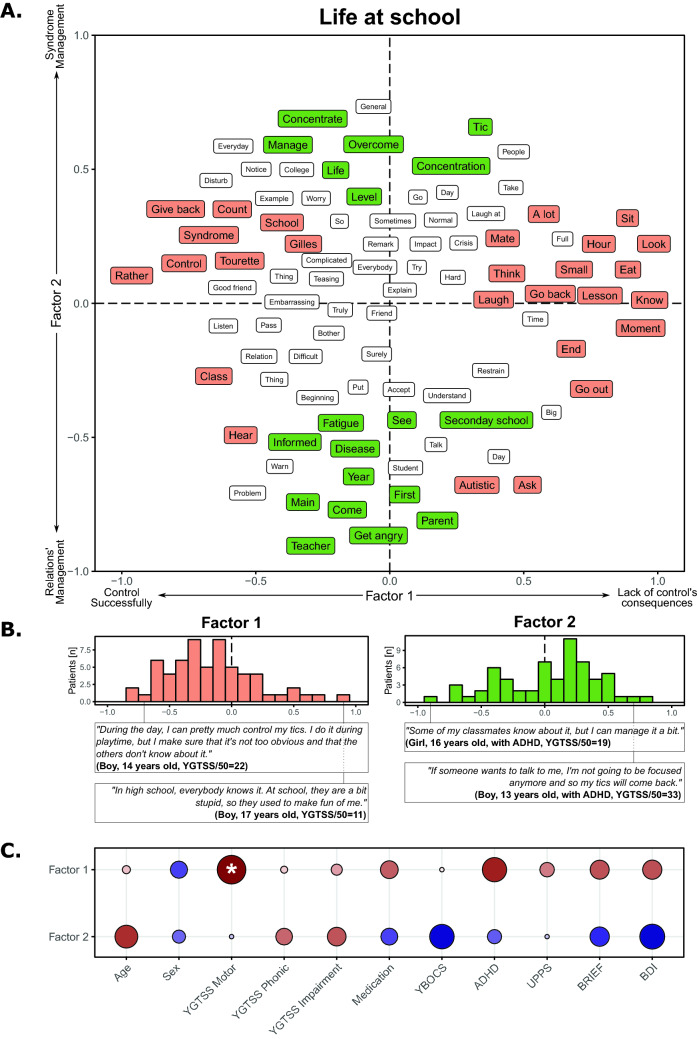
Results on the daily living at school corpus

The life at school corpus was composed of 8,818 words. The first factor of the FCA described the control of tics, ranging from successful control (negative score) to the consequences of a lack of control (positive score; see Fig. 3A). For this factor, most of the patients (69%) referred more to successful control (*χ*_(1)_ = 9.29, *p* = 0.0023; Fig. 3B) and we found a positive correlation with motor tic severity (*t*_(60)_ = 2.107, *p* = 0.039, *r* = 0.262; Fig. 3C). The second factor referred to the management of relations with others (negative score) or of the syndrome itself (positive score; Fig. 3A). Most of the patients (66%) were more focused on management of the syndrome (*χ*_(1)_ = 6.45, *p* = 0.011; Fig. 3B). No demographic or clinical correlation was found with this factor (Fig. 3C).

Panel A shows the results of the factorial analysis for the first two factors. Words in orange correspond to words with a significant involvement on the first factor, while words in green correspond to words with a significant involvement on the second factor. Panel B highlights the position of the TS patients on the first factor (left) and on the second factor (right), and the sentences of the most typical individuals with TS. Panel C shows the correlations between the positions of factor 1 (top) and factor 2 (bottom) and the demographic and clinical variables. Red colors correspond to positive correlations, while blue colors correspond to negative correlations. The size of the point and the color intensity indicate the strength of the correlations. ‘*’ indicates a significant correlation. Note that a word could be present multiple times due to the translation from the original French results to English.

### Corpus 4: interactions with strangers

**Fig. 4:**
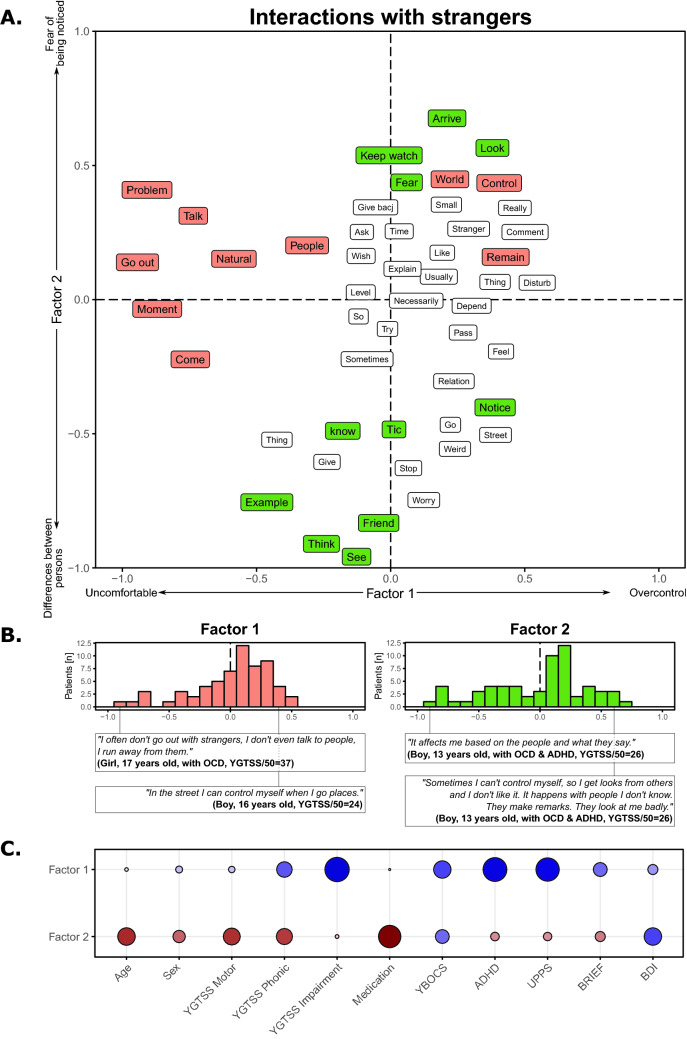
Results on the relation with strangers corpus

The interactions with strangers’ corpus were composed of 4671 words. The first FCA factor described being uncomfortable with strangers (negative score) or being in an overcontrol situation (positive score; Fig. 4A) without imbalance between these two parts (*χ*_(1)_ = 3.16, *p* = 0.075; Fig. 4B). No significant correlation was found with demographic or clinical variables (Fig. 4C). The second factor ranged from the differences between familiar and unfamiliar persons (negative scores) to the fear of being noticed (positive score; Fig. 4A), with an equivalent distribution (χ_(1)_ = 2.32, *p* = 0.12; Fig. 4B). There was no significant demographic or clinical correlations (Fig. 4C).

Panel A shows the results of the factorial analysis for the first two factors. Words in orange correspond to words with a significant involvement on the first factor, while words in green correspond to words with a significant involvement on the second factor. Panel B highlights the position of the TS patients on the first factor (left) and on the second factor (right), and the sentences of the most typical individuals with TS. Panel C shows the correlations between the positions of factor 1 (top) and factor 2 (bottom) and the demographic and clinical variables. Red colors correspond to positive correlations, while blue colors correspond to negative correlations. The size of the point and the color intensity indicate the strength of the correlations. ‘*’ indicates a significant correlation. Note that a word could be present multiple times due to the translation from the original French results to English.

### Corpus 5: future expectations

**Fig. 5:**
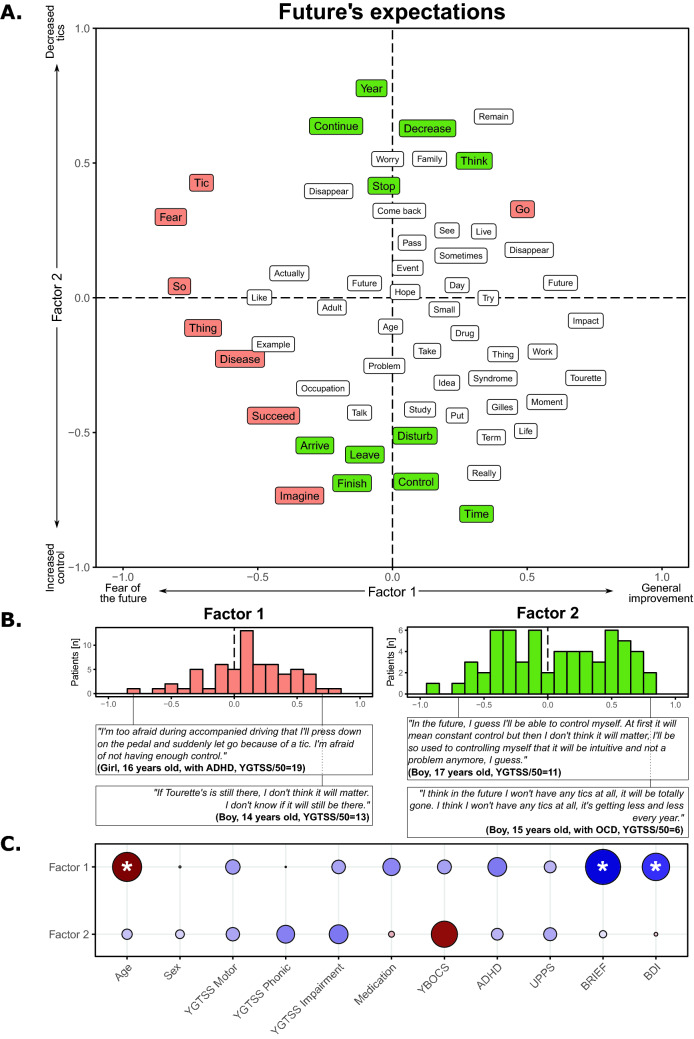
Results on the expectations regarding future corpus

The future expectations’ corpus was composed of 5,290 words. The first factor highlighted a range from patients with a fear of the future (negative score) to patients who expected a general improvement (positive score; Fig. 5A). A larger number of individuals with TS (69%) had a positive score (*χ*_(1)_ = 9.29, *p* = 0.002; Fig. 5B). In addition, this factor was positively correlated with the patients’ age (*t*_(60)_ = 2.137, *p* = 0.037, *r* = 0.266) and negatively with their BRIEF (*t*_(60)_ = − 2.586, *p* = 0.012, *r* = − 0.317) and BDI scores (*t*_(60)_ = − 2.008, *p* = 0.049, *r* = − 0.251; Fig. 5C). The second factor suggested two types of improvement, an increase in control over tics (negative score) or a decrease in tics (positive score; Fig. 5A), with an equivalent distribution between these two topics (*χ*_(1)_ = 0.06, *p* = 0.79; Fig. 5B), and without any significant correlation with clinical scores (Fig. 5C).

Panel A shows the results of the factorial analysis for the first two factors. Words in orange correspond to words with a significant involvement on the first factor, while words in green correspond to words with a significant involvement on the second factor. Panel B highlights the position of the TS patients on the first factor (left) and on the second factor (right), and the sentences of the most typical individuals with TS. Panel C shows the correlations between the position of factor 1 (top) and factor 2 (bottom) and the demographic and clinical variables. Red colors correspond to positive correlations, while blue colors correspond to negative correlations. The size of the point and the color intensity indicate the strength of the correlations. ‘*’ indicates a significant correlation. Note that a word could be present multiple times due to the translation from the original French results to English.

### Correlations among corpuses

Our correlation analyses revealed only one significant association among corpora. This correlation was found between the second factor of the main difficulty corpus (from the cost of management to the fear of disturbing others) and the second factor of the interactions with strangers corpus (from differences between persons to fear of being noticed; *t*_(60)_ = 3.195, *p* = 0.002, *r* = 0.381). In other words, TS patients with the greatest fear of disturbing others also have the greatest fear of being noticed by strangers, and patients with the cost of managing symptoms as one of the main difficulties also discussed a strong difference between familiar and unfamiliar persons.

## Discussion

The analysis of interviews of adolescents with TS revealed several important themes. Interestingly, some of them were directly related to the severity of tics, while others were disconnected from clinical features. In general, we observed that (i) social stigma/perceived stigma was a pervasive topic, (ii) that tic severity was especially related to difficulties at school, (iii) ADHD was related to a higher fear of receiving remarks from others, (iv) while OCD was more related to a higher cost of control over symptoms, and (v) that some assessments performed by their parents are aligned with the patients’ worries.

The main limitation of this study was related to the relatively short duration of the interviews and their restriction to our main domains of interest. If this limitation does not detract from the main conclusions of our study, longer interviews with more diversified topics could have led to the identification of more varied results. In addition, we decided to not apply any statistical correction for the correlations we achieved with the demographic and clinical variables, since none of them reached the threshold of significance after such a correction. Therefore, future studies with a larger sample of participants will be valuable to validate or not the conclusions based on these variables.

### Social stigma is the most common and recurrent issue faced by TS patients

Our results highlighted the strong impact of stigma/perceived stigma. This aspect was found in the two factors related to the main difficulty (i.e., others’ eyes and fear of disturbing others), in relations with family (i.e., the fact that some family members could laugh about it), in school (i.e., the management of social relationships), and during interactions with strangers (i.e., the fear of being noticed). In addition, our sole significant inter-corpus correlation was found between the fear of being noticed in general (main difficulty corpus) and the fear of being noticed by strangers in particular. A systematic review focused on stigma in TS revealed some interesting findings which could contribute to explain the omnipresence of stigma we found [10]. First, most teachers and peers only have basic knowledge about TS [24, 25], explaining why the social stigma surrounding TS is essentially said to stem from a lack of understanding and acceptance from both schoolteachers and peers [26, 27]. Second, most normally developing children have a negative view of individuals living with tics [28, 29]. Third, most of the patients’ parents reported that their child suffered from discrimination at school [7]. Fourth, most of the patients experienced self-degrading comments as a consequence of their syndrome [30]. Altogether, these points could contribute to considerable difficulties, especially at school, which could lead to a higher probability of failure or even lead to leaving school. This also suggests that TS must be considered more from a social point of view. As already suggested, improving general knowledge about TS by decreasing the stereotypes surrounding it could be a way to decrease the social stigma induced by this syndrome [10].

### Impact of tic severity on daily life at school

Several aspects of the TS patients’ daily lives were directly affected by clinical severity. The first was related to the severity of motor tics and the patients’ ability to control tics, especially at school. Indeed, control of tics at school corresponded to the first factor of our FCA, meaning that this was the most relevant and recurrent topic among our patients. Although most of them could control their tics, the consequences of a lack of control (more present in patients with more severe motor tics) were more often discussed, including the fear of being mocked (“*laughed at*”) or to failing in their schooling (“*lessons*”). This result is in line with previous studies, which report that tics contribute to difficulties at school, including difficulties reading [26], writing [6], concentrating [27], and interacting socially [30]. Usually, this effect could be specifically (or exclusively) related to school, since it is known that activities related to school tend to increase the manifestation of tics [31]. Because of the importance of school in children’s growth and development, the conflicts and tensions that arise in the classroom can impact young people with TS in many ways, which in some cases results in school avoidance or even refusal [27, 32].

### Impact of ADHD and OCD comorbidities on daily living and risk of depression

The presence of comorbid ADHD and OCD was found to be related to the most significant difficulties. Specifically, ADHD was related to a higher fear of receiving remarks from others, while OCD was more related to a higher cost of control over symptoms. Interestingly, the analysis of the main difficulty also revealed a relationship with a higher score of depression. In addition, this higher depression score was also found for patients with a more pessimistic view of their future. These results are in line with previous studies which highlighted that ADHD and OCD comorbidities in TS were directly related to a higher probability of experiencing negative life events and of developing depression [33]. For example, ADHD was already related to impaired social and emotional functioning and increased loneliness [34, 35]. Taken together, these results emphasize that patients who have to control both their tics and symptoms of comorbidities will suffer from general fatigue and lack of energy, leading to hopelessness regarding their future.

### Parents’ perception as a reflection of patients’ worries

Some aspects of daily life were found to be related to executive functioning assessed by the patients’ parents (i.e., BRIEF). This point is especially interesting, since it was found for one aspect of the family living (i.e., talking about TS with other members of the family) and the future expectations of the patients (i.e., the fear about a lack of improvement during adulthood). This result highlighted the family members’ role, which could strongly vary from the patients’ point of view. While some report that parental over-supervision and pressure lead to an increase in tic expression and consequently to familial conflicts and tensions [26], others report that parents and family members are the most supportive and understanding of tics and comorbid symptoms [26, 36, 37]. Yet even then, parents can experience feelings of guilt [38], self-blame [32], and anxiety regarding their child’s prospective wellbeing [26, 39]. Conflicts at home most commonly result from maladaptive and disruptive behaviors between the child with TS and family members [40], while disagreements in public spheres can arise from parents’ strong desire for their children to suppress and control their tics to display an appropriate behavior and avoid attracting attention [26, 32]. Therefore, even if difficult, the parents’ position is of significant importance as their evaluation of their children is a powerful reflection of the future expectations of the youngest patients.

## Conclusion

To the best of our knowledge, this study is the first to investigate the impact of TS on the daily life of adolescents living with TS using interviews and a statistical text mining methodology. By exploring several domains of their daily life, we reported the pervasive position of social stigma, but also some difficulties directly related to clinical features. Taken together, we can conclude that going through adolescence with TS is essentially difficult from a social standpoint and is more costly for patients who are also living with ADHD or OCD comorbidities. As a recommendation, we suggest trying to both increase the general knowledge about TS and decrease the stereotypes surrounding it, at least at school.

Finally, from a methodological standpoint, this study also demonstrated the feasibility of analyzing a high volume of textual data to represent meaningful information which is generally under-considered. This approach allows data to be obtained in a natural and unanticipated way and to highlight several points without any a priori.

Future interesting studies based on text mining could assess the standpoint of peers on TS (e.g., family members, teachers, and students), the speech of both younger and adult patients, or the correlates of quality of life as measured by self-assessments.

### Supplementary Information

Below is the link to the electronic supplementary material.

Supplementary file1 (DOCX 453 KB)

## Data Availability

Available from the corresponding author upon reasonable request.
