# Supplementary material for: How does Tourette syndrome impact adolescents’ daily living? A text mining study
Source: Eur Child Adolesc Psychiatry. 2022 Dec 2;32(12):2623–35. doi: 10.1007/s00787-022-02116-1 (PMC10682273; doi:10.1007/s00787-022-02116-1)
Supplement: Supplementary file 1 — Supplementary file1 (DOCX 453 KB) [file 787_2022_2116_MOESM1_ESM.docx]

**Supplementary material**

**Table.sup.1 Descriptive data and statistics of the 62 TS patients included in the study.**

|  | **N / Mean ± SD / %** | **Range** |
| --- | --- | --- |
| Gender (M / F) | 47 / 15 | - |
| Age | 15.8 ± 1.8 | [13 – 18] |
| YGTSS – 50 (Motor) | 11.3 ± 4 | [0 – 20] |
| YGTSS – 50 (Verbal) | 7.3 ± 4.9 | [0 – 18] |
| YGTSS – 50 (Total) | 18.5 ± 7.4 | [5 – 37] |
| ASRS | 35 ± 11.8 | [13 – 59] |
| Y-BOCS | 13.4 ± 8.4 | [0 – 33] |
| BDI | 10.6 ± 8.9 | [0 – 44] |
| UPPS | 112.3 ± 19.1 | [58 – 149] |
| BRIEF | 71.1 ± 34.3 | [3 – 149] |
| Medication (%) | 50 % | - |
| ADHD (%) | 30.6 % | - |
| OCD (%) | 25.8 % | - |

ADHD: Attention-Deficit Hyperactivity Disorder; ASRS: ADHD Self-Report Scale; BDI: Beck Depression Inventory; BRIEF: Behavior Rating Inventory of Executive Function; F: Female; M: Male; OCD: Obsessive-Compulsive Disorder; SD: Standard deviation; UPPS: Urgency, Premeditation, Perseverance, Sensation Seeking, Impulsive Behavior Scale; Y-BOCS: Yale-Brown Obsessive Compulsive Scale; YGTSS: Yale Global Tic Severity Scale.


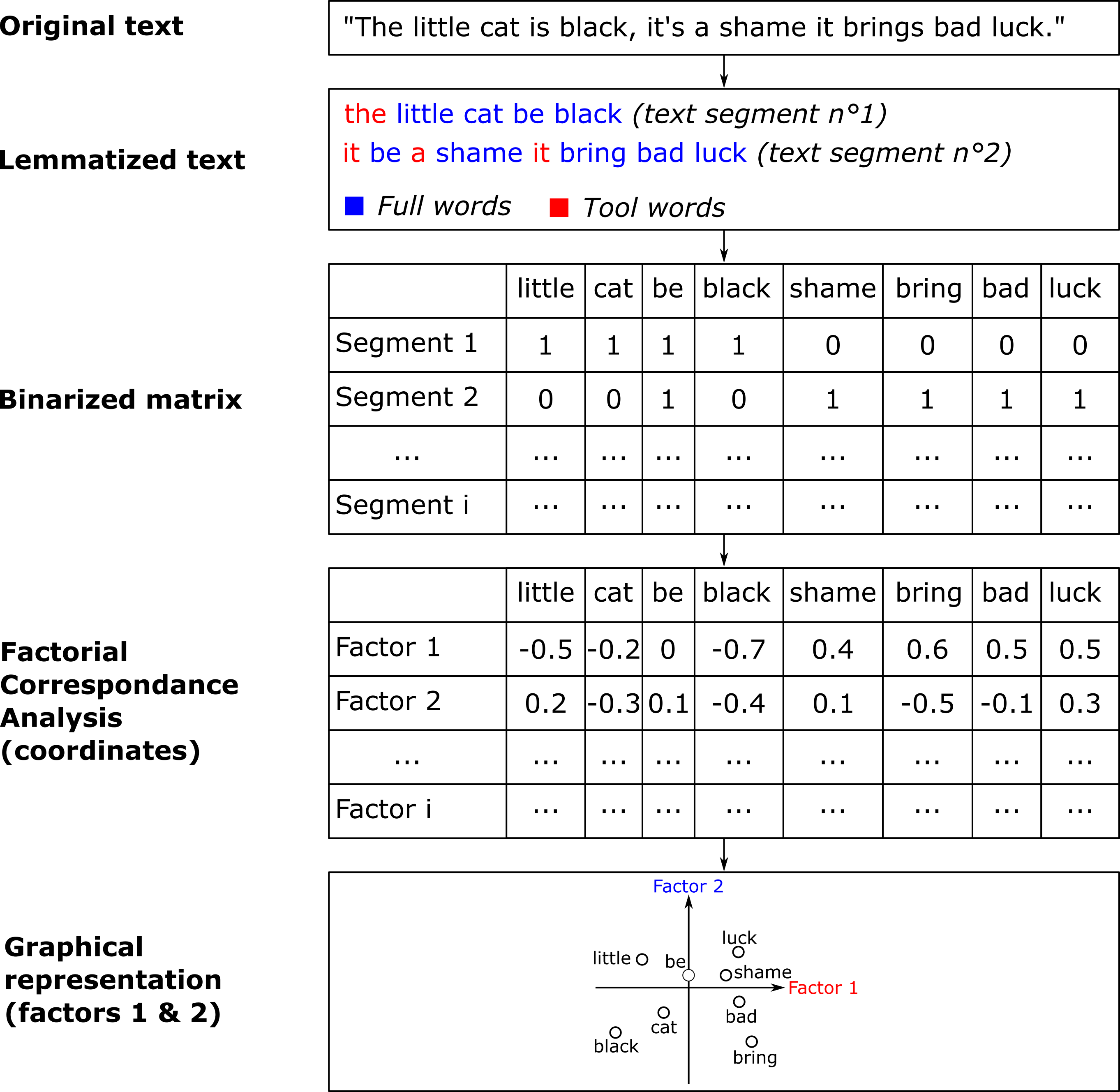


**Figure.sup.1 Illustration of the FCA process.**

The original text is first lemmatized in order to group together the inflected forms of a word in their simplest form. Then, words are categorized into two subcategories: “full words” (e.g., verbs, nouns, adjectives) and “tool words” (e.g., pronouns, determents) in order to only consider full words in the analyses. This text is then transformed into a binarized matrix with texts segments as rows and words as columns. The FCA is then applied to this matrix to obtain the coordinates of each word for each factor. Lastly, the first two factors are represented by a 2-dimensional graphic.
